# Strain-encoded cardiac magnetic resonance during high-dose dobutamine stress testing for the estimation of cardiac outcomes. Comparison to clinical parameters and conventional wall motion readings

**DOI:** 10.1186/1532-429X-13-S1-O6

**Published:** 2011-02-02

**Authors:** Gitsios Gitsioudis, Stephanie Lehrke, Nina Riedle, Sebastian J Buss, Evangelos Giannitsis, Nael F Osman, Hugo A Katus, Grigorios Korosoglou

**Affiliations:** 1 University Clinic of Heidelberg, Heidelberg, Germany

## Introduction

DS-MRI is a well established modality for the diagnostic classification and the risk stratification of patients with coronary artery disease (CAD). However, currently the detection of inducible ischemia by DS-MRI is based on the assessment of cine images, which is subjective and depends on the experience of the readers. In this regard, the human eye primarily tracks the radial displacement of the myocardium with cine-images, which is less sensitive than circumferential and longitudinal components for the detection of myocardial dysfunction.

## Purpose

To determine the prognostic value of Strain-Encoded-MRI (SENC) during high-dose dobutamine stress cardiac magnetic resonance imaging (DS-MRI) compared with conventional wall motion analysis.

## Methods

320 consecutive patients with suspected or known CAD underwent DS-MRI, using a standard protocol in a 1.5T MR-scanner. Wall motion and SENC were assessed at baseline and during stress, and outcome data including cardiac deaths, nonfatal myocardial infarctions (‘hard events’) and revascularization procedures performed >90 days after the MR-scans were collected.

## Results

**Figure 1:**
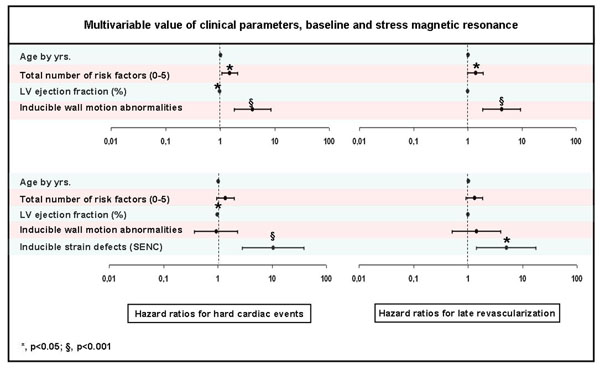
Mutlivariable value of clinical parameters, baseline and stress magnetic resonance

35 hard events occurred during a 28±9 month follow-up period, including 10 cardiac deaths and 25 nonfatal myocardial infarctions, while 32 patients underwent coronary revascularization. Using multivariable Cox proportional-hazards models, atherogenic risk factors and inducible wall motion abnormalities (WMA) were independently associated with hard events (HR=1.5 (95%CI=1.1-2.1), p=0.02 for the total number risk factors and HR=3.9 (95%CI=1.8-8.6) for inducible WMA, p<0.001, respectively). When inducible strain defects were additionally considered in the model, however inducible WMA were no longer predictive, and SENC yielded the strongest independent association with cardiac outcome (HR of 10.5 (95%CI=2.9-38.5), p<0.001) (Figure 1). Furthermore, quantification analysis showed that a cut-off value of <1.64 for strain rate reserve was highly predictive for future hard cardiac events (AUC=0.82, 95% CI=0.78-0.86; negative predictive value=98%).

## Conclusions

Strain-Encoded-MRI aids the accurate identification of patients at high risk for future cardiac events and revascularization procedures, beyond the assessment of conventional atherogenic risk factors and inducible WMA on cine-images.

